# The Design, Synthesis and Mechanism of Action of Paxlovid, a Protease Inhibitor Drug Combination for the Treatment of COVID-19

**DOI:** 10.3390/pharmaceutics16020217

**Published:** 2024-02-02

**Authors:** Miklós Bege, Anikó Borbás

**Affiliations:** 1 Department of Pharmaceutical Chemistry, University of Debrecen, Egyetem tér 1, 4032 Debrecen, Hungary; bege.miklos@euipar.unideb.hu; 2 HUN-REN-DE Molecular Recognition and Interaction Research Group, University of Debrecen, Egyetem tér 1, 4032 Debrecen, Hungary; 3 Institute of Healthcare Industry, University of Debrecen, Nagyerdei krt 98, 4032 Debrecen, Hungary; 4 National Laboratory of Virology, University of Pécs, Ifjúság útja 20, 7624 Pécs, Hungary

**Keywords:** viral proteases, SARS-CoV-2, non-structural protein (NSP), main protease (M^pro^), 3CL protease, nirmatrelvir/ritonavir, electrophilic warhead, covalent inhibitor, booster, drug–drug interactions

## Abstract

The COVID-19 pandemic, caused by severe acute respiratory syndrome coronavirus 2 (SARS-CoV-2), has presented an enormous challenge to health care systems and medicine. As a result of global research efforts aimed at preventing and effectively treating SARS-CoV-2 infection, vaccines with fundamentally new mechanisms of action and some small-molecule antiviral drugs targeting key proteins in the viral cycle have been developed. The most effective small-molecule drug approved to date for the treatment of COVID-19 is Paxlovid^TM^, which is a combination of two protease inhibitors, nirmatrelvir and ritonavir. Nirmatrelvir is a reversible covalent peptidomimetic inhibitor of the main protease (M^pro^) of SARS-CoV-2, which enzyme plays a crucial role in viral reproduction. In this combination, ritonavir serves as a pharmacokinetic enhancer, it irreversibly inhibits the cytochrome CYP3A4 enzyme responsible for the rapid metabolism of nirmatrelvir, thereby increasing the half-life and bioavailability of nirmatrelvir. In this tutorial review, we summarize the development and pharmaceutical chemistry aspects of Paxlovid, covering the evolution of protease inhibitors, the warhead design, synthesis and the mechanism of action of nirmatrelvir, as well as the synthesis of ritonavir and its CYP3A4 inhibition mechanism. The efficacy of Paxlovid to novel virus mutants is also overviewed.

## 1. Introduction—Viral Proteases as Drug Targets

The genetics and reproduction of viruses differ significantly from what we are used to in eukaryotes in many respects. One important difference is that many viruses, including retroviruses, herpesviruses, flaviviruses and coronaviruses, do not encode functional proteins that are synthesized individually, but rather one or two large polyproteins that are then cleaved by viral proteases into functional proteins [1]. Proteases are a subgroup of hydrolases, enzymes that catalyze hydrolytic reactions. There are many mechanisms of proteolysis; a common method is the use of a nucleophilic group, generated from the side chain of serine (serine proteases) or cysteine (cysteine proteases), which can perform a nucleophilic attack on the partially positively polarized carbonyl carbon atom of the peptide bond. In aspartic proteases, a water molecule bound to aspartic acid in the active site of the enzyme acts as a nucleophile. In the case of serine and cysteine proteases, the nucleophile is generated by the amino acids in the active site of the enzyme. A common system for this is the so-called catalytic dyad or catalytic triad, which consists of two or three amino acids [1,2]. One of the three amino acids that make up the catalytic triad carries an acidic side chain, e.g., aspartic acid (Asp), the other carries an alkaline side chain, e.g., histidine (His), and the nucleophilic group is formed from the third amino acid, which is serine (Ser) or cysteine (Cys).

**Scheme 1:**
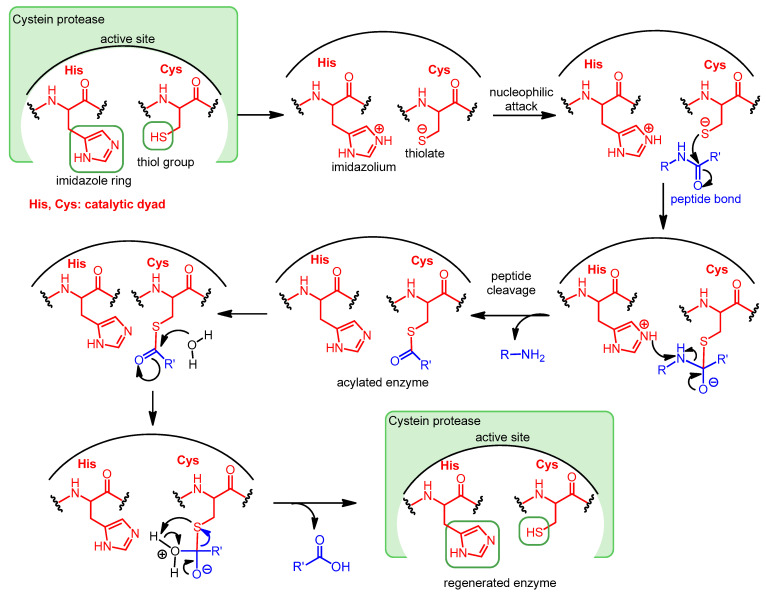
Catalytic mechanism of Cys proteases operating with a catalytic dyad.

In some cysteine hydrolases, histidine and cysteine form a catalytic dyad in the active site; in such enzymes, the role of the third amino acid, Asp, is played by an activated water molecule (Scheme 1). In the first step of the enzyme’s catalytic mechanism, the imidazole ring of histidine as a base deprotonates the thiol group of cysteine, forming a thiolate–imidazolium ion pair. Next, the thiolate group performs a nucleophilic attack on the carbonyl C atom of the peptide bond of its substrate. As a result, the peptide bond is cleaved, the amine terminus of the peptide fragment (R-NH_2_) is released, while the acyl part forms a thioester with the cysteine, and the histidine is reestablished to its deprotonated form. Finally, the thioester bond of the acylated enzyme is hydrolyzed by an activated water molecule to generate a carboxylic acid group on the remaining substrate fragment (R’-COOH), regenerating the free enzyme.

Viral proteases play a key role in viral replication for all positive single-stranded RNA viruses and some DNA viruses, such as herpesviruses. To treat infections caused by these viruses, proteases are considered excellent drug targets [3]. Protease inhibitors are now routinely used in antiviral therapy for human immunodeficiency virus (HIV) and hepatitis C virus (HCV) infections.

The pathogen responsible for the outbreak of the COVID-19 pandemic, severe acute respiratory syndrome coronavirus 2 (SARS-CoV-2), is an enveloped β-coronavirus. It belongs to the family of positive single-stranded RNA viruses and, as such, also encodes proteases. SARS-CoV-2 main protease (M^pro^), also known as 3-chymotrypsin-like protease (3CLpro) or nonstructural protein 5 (NSP5), is a cysteine hydrolase that plays an essential role in the SARS-CoV-2 life cycle. M^pro^ is a homodimer with each monomer consisting of three distinct domains (I, II and III). Both monomers contain a catalytic site at the interface of domains I and II, but only one active site is functional in the dimeric form. Domain III is required for homodimerization, which is critical to the enzyme’s catalytic activity. The enzyme has six substrate binding sub-pockets in the active site, sub-pockets S1, S2 and S4 in the protein cavity, while sub-pockets S1’, S3 and S5 are located on the surface of the protein. The active site of the enzyme contains a catalytic dyad consisting of Cys145 and His41, and a catalytic water molecule H-bonded to His41 [4,5,6].

Since SARS-CoV-2 M^pro^ plays a key role in viral replication by cleaving viral polyproteins, inhibition of its catalytic activity represents an attractive therapeutic approach for the treatment of COVID-19. In addition, M^pro^ has two properties that make it an ideal target for antiviral drugs. First, its recognized sequence is Leu-Gln-Ser-Ala-Gly, and it cleaves the peptide chain after a glutamine (Gln) unit; since there are no known human Cys proteases that cleave the protein after Gln, the proteolytic action of SARS-CoV-2 M^pro^ can be specifically inhibited without inhibiting human proteases. Second, unlike spike protein, M^pro^ is a highly conserved protein, mutations in this protein could be fatal to the virus, which reduces the risk of developing drug resistance [5,6].

Nirmatrelvir is a newly developed potent inhibitor of the main protease of SARS-CoV-2. However, due to its rapid metabolism by the cytochrome CYP3A4 enzyme, it is not effective on its own in vivo. Nirmatrelvir became suitable for therapeutic use in com bination with ritonavir, originally designed as a HIV protease inhibitor, which can sufficiently increase the bioavailability of nirmatrelvir by inhibiting the CYP3A4 enzyme. The nirmatrelvir–ritonavir combination was developed by Pfizer and marketed under the brand name Paxlovid^TM^ as an oral drug for the treatment of COVID-19.

Since the approval of Paxlovid for the emergency treatment of COVID-19 in 2021, many reviews have been published focusing primarily on the drug’s effectiveness, safety, side effects and drug–drug interactions [7,8,9,10,11,12]. In this tutorial review, we give a medicinal chemistry overview of the two pharmacologically active components of Paxlovid, focusing on the structural similarities of currently used protease inhibitor antivirals, the role of the warhead in the mechanism of action of nirmatrelvir, as well as the details of the booster effect of the ritonavir. We also briefly discuss drug–drug interactions and Paxlovid’s effectiveness against new virus variants.

## 2. Protease Inhibitors as Antivirals

### 2.1. Protease Inhibitor Drugs for the Treatment of HCV and HIV Infections

**Figure 1:**
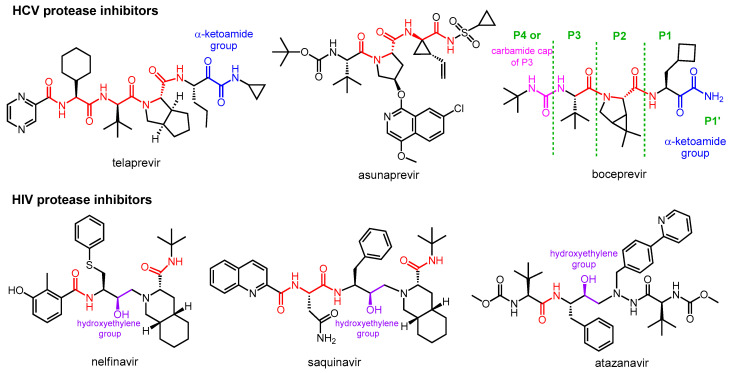
Structures of viral protease inhibitor drugs.

The two main groups of protease inhibitors (PIs) currently used in medicine are HIV and HCV protease inhibitors (Figure 1). Hepatitis C virus (HCV) is a small RNA virus that causes hepatic diseases. HCV protease inhibitors, such as asunaporevir, telaprevir, and boceprevir, target the NS3/4A serine protease of the virus (NS stands for “nonstructural” in the name of viral proteins, indicating that the given protein is not a structural protein) [13,14]. The structures of these protease inhibitors appear to be very different, but all contain at least one peptide (amide) bond (highlighted in red in Figure 1). This is necessary because these inhibitors act by binding to the active site of the enzyme, so they must have a chemical structure similar to the natural peptide substrate, so the inhibitors are peptidomimetics. Some of the inhibitors contain a “warhead” group, which reacts with the enzyme and binds covalently to the active site, thereby inactivating the protease. It is important to note that although inhibitor–protease binding is usually covalent, the inhibition is reversible. The warheads used in the case of serine protease include, for example, an α-ketoamide, boronic acid or α-keto acid group. To learn about the general structure of protease inhibitors, let us take a closer look at boceprevir. In the commonly used nomenclature of protease inhibitors, the positions from the cleavage site towards the C-terminal of the molecule are numbered P1′, P2′, P3′, etc., while the groups towards the *N*-terminal are P1, P2, P3, etc. [15]. Accordingly, there is an α-ketoamide warhead in the P1 position of boceprevir to ensure the covalent inhibitory effect. Other parts of the molecule serve to bind to the enzyme with secondary bonds. The P3 position is “capped” with a carbamide type group.

HIV protease inhibitors are used against the human immunodeficiency virus (HIV), which causes Acquired Immune Deficiency Syndrome (AIDS). HIV protease is an aspartic acid protease that cleaves the peptide bond between a phenylalanine and a proline. Its inhibitors, such as nelfinavir, saquinavir, atazanavir, etc., are peptidomimetics that contain a non-cleavable hydroxyethylene group [16,17]. HIV PIs are used in highly efficient antiretroviral therapy, which is the standard protocol for treating HIV infection, converting it from a fatal disease to a chronic infection.

### 2.2. Development and Mechanism of Action of Nirmatrelvir

The structure of nirmatrelvir (PF-07321332) [4] from Pfizer can be traced back to a previous Pfizer compound, PF-00835231 [18], which was generated to inhibit the main protease of SARS-CoV-1. The zoonotic coronavirus SARS-CoV-1 emerged in China in 2002 and caused an epidemic leading to ~8000 cases and almost 800 confirmed deaths [19]. Due to the high similarity (~96% sequence homology) between the main proteases of the two viruses, PF-00835231 can also inhibit SARS-CoV-2 M^pro^ [4,18].

**Figure 2:**
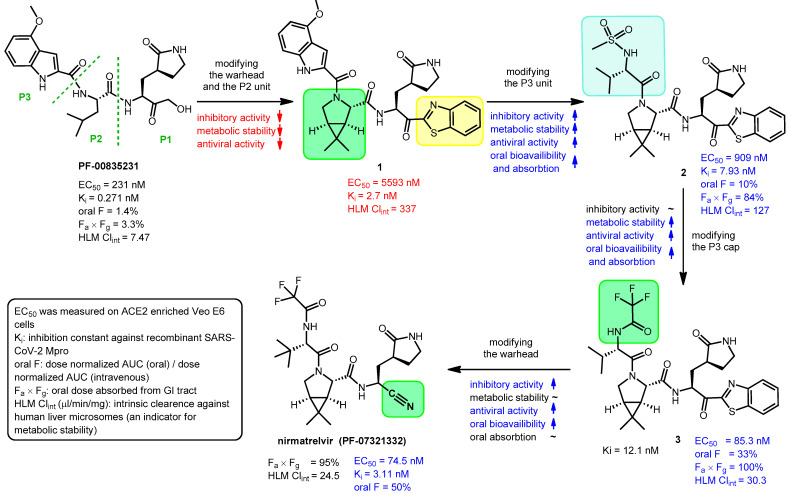
Development of nirmatrelvir. EC_50_: half-maximal effective concentration, oral F: oral bioavailability (oral fraction) in rat, AUC: area under curve, GI tract: gastrointestinal tract.

The problem with PF-00835231 is that it has very low oral absorption. A development process was started by Pfizer to solve the problem (Figure 2) [4,6,20]. One way to increase the oral bioavailability of a molecule is to reduce the number of hydrogen bond donor groups (HBD) [21]. Therefore, the α-hydroxymethyl ketone warhead was changed to benzothiazol-2-yl ketone (highlighted in yellow). Furthermore, the P2 unit was replaced by a pyrrolidine ring (highlighted in green in **1**) to get rid of the N–H group, which is an HBD group, to give compound **1**. However, these changes eliminated the possibility of H-bonding with amino acid Gln-189 of M^pro^, thereby weakening the binding to the target enzyme, which reduced the inhibitory activity. The indole component P3 was replaced with a smaller acyclic sulfonamide unit (highlighted in blue) to fit into the S3 pocket of M^pro^, thereby increasing the binding affinity to the enzyme, which increased the inhibitory effect. The antiviral activity of compound **2** obtained through the changes was indeed better than that of compound **1**, and its oral bioavailability was higher than that of compound PF-00835231. Subsequently, the P3 cap was replaced with a trifluoroacetamide group (highlighted in green in **3**), which further improved antiviral activity, as well as increased metabolic stability and oral pharmacokinetics. Finally, the introduction of the nitrile warhead at the P1′ position (highlighted in green in nirmatrelvir) further enhanced the antiviral activity and oral bioavailability. An additional advantage of the nitrile warhead was that it facilitated the synthesis, since the nitrile-containing compound was more soluble and less prone to epimerization [4].

During the development process, the activity of the compounds was compared with enzyme inhibitory (K_i_) and anti-SARS-CoV-2 (EC_50_) measurements, and for their pharmacokinetic characterization, oral bioavailability (oral F) and metabolic stability tests were performed [4]. Oral bioavailability was assessed in rats and metabolic stability was assessed by measuring intrinsic clearance against human liver microsomes (HLM Cl_intr_). The results of these tests are summarized in Figure 2.

**Figure 3:**
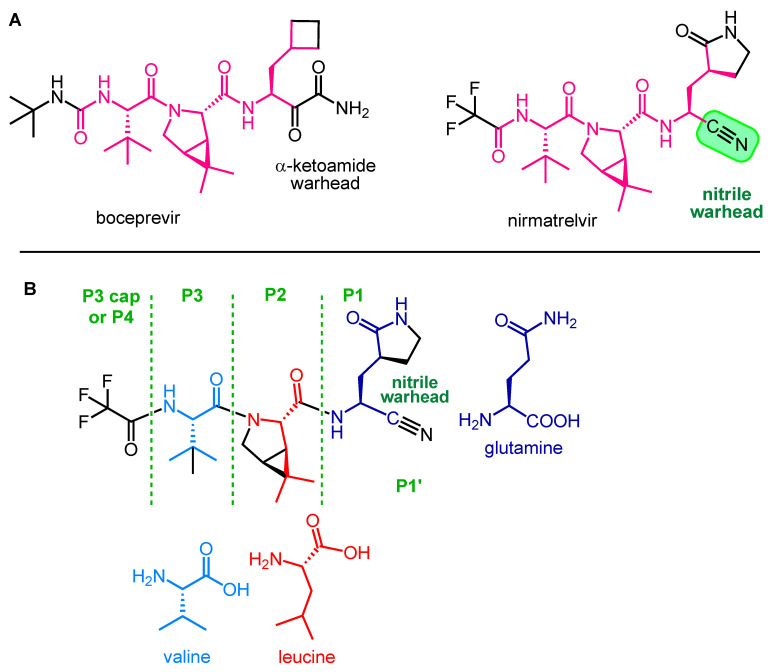
Structural similarity between boceprevir and nirmatrelvir (**A**) and main structural elements of nirmatrelvir (**B**).

Nirmatrelvir (PF-07321332) is an orally administered covalent inhibitor of SARS-CoV-2 M^pro^. As shown in Figure 3A, it has a high structural similarity to boceprevir, but their warheads are different.

The main structural elements of nirmatrelvir are depicted in Figure 3B. The warhead of the molecule is the nitrile group in the P1′ position, which covalently reacts with the enzyme, while the rest of the molecule, mimicking the natural recognition sequence of M^pro^, the tripeptide Val-Leu-Gln (valine-leucine-glutamine), ensures that nirmatrelvir fits into the active site of the enzyme and binds there with secondary bonds [22]. The P1 unit is a Gln analog with a cyclic (γ-lactam) side chain that can form H-bonds. It is an advantageous modification because the amide group of native Gln can react intramolecularly with certain types of warheads, rendering the molecule ineffective. This cyclic moiety is more rigid than the native Gln side chain, which may be beneficial in terms of binding to the target enzyme [23]. Furthermore, the modification of Gln into a cyclic derivative also helps the synthesis. The P2 group is a dimethylcyclopropyl proline (DMCP) and is a leucine analog that binds primarily to the enzyme at the S2 position through lipophilic interactions. The P3 residue is a tertiary leucine that mimics valine. The *N*-terminal is capped with a trifluoroacetyl group [4,24].

The nitrile warhead plays a key role in the protease inhibitory mechanism of nirmatrelvir. It is worth noting that the nitrile group is also used in other drugs (e.g., vildagliptin) and drug candidates (e.g., Cbz-A VLQ-CN) to target serine and cysteine peptidases. Although nitrile is less reactive compared to other warheads (e.g., aldehydes), which can be a disadvantage; however, on the other hand, it provides better selectivity and metabolic stability [23].

**Scheme 2:**
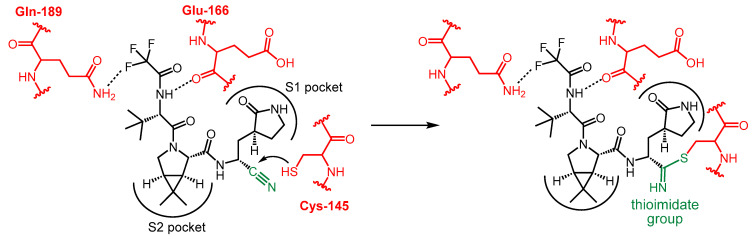
Mechanism of action of nirmatrelvir.

The covalent interaction between nirmatrelvir and SARS-CoV-2 main protease is shown in Scheme 2. The P1′ nitrile group forms a thioimidate bond with the Cys-145 thiol functional group of M^pro^ through a Pinner-like reaction. Since the thiol group of Cys-145 is essential for catalyzing the hydrolysis of peptide bonds, the function of the enzyme is blocked [20,25].

### 2.3. Synthesis of Nirmatrelvir

**Scheme 3:**
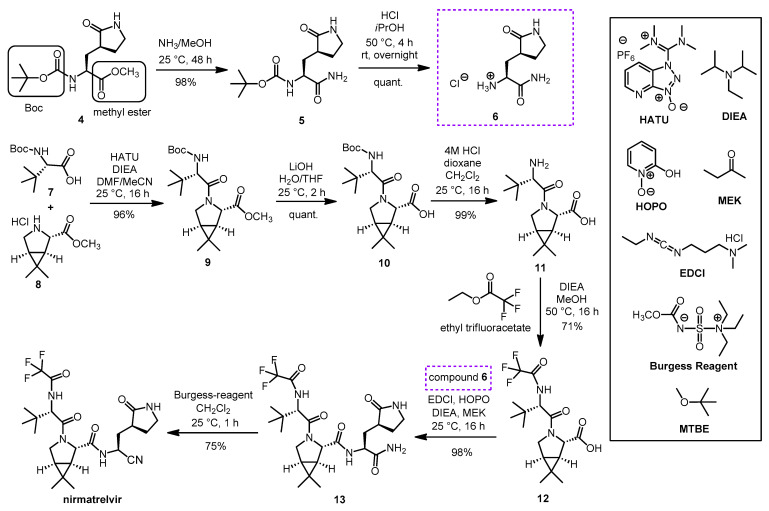
Synthesis of nirmatrelvir. (HATU: *O*-(7-azabenzotriazol-1-yl)-*N*,*N*,*N’*,*N’*-tetramethyluronium hexafluorophosphate, DIEA: diisopropyl-ethylamine, HOPO: 2-hydroxypyridine 1-oxide, MEK: methyl ethyl ketone, EDCl: 1-[3-(dimethylamino)propyl]-3-ethylcarbodiimide hydrochloride, DMF: *N*,*N*-dimethylformamide, THF: tetrahydrofuran, MTBE: methyl *tert*-butyl ether.).

**Scheme 4:**
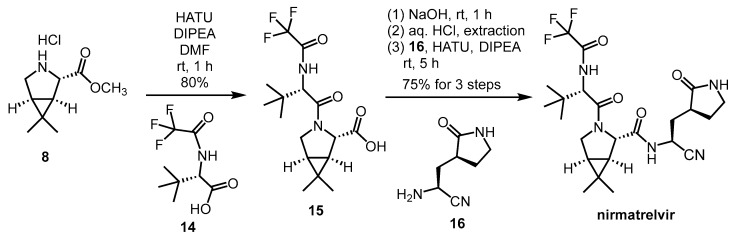
Alternative synthesis of nirmatrelvir. (HATU: *O*-(7-azabenzotriazol-1-yl)-*N*,*N*,*N’*,*N’*-tetramethyluronium hexafluorophosphate, DIPEA = DIEA: diisopropyl-ethylamine).

A synthesis route of nirmatrelvir developed by Pfizer involved the preparation and coupling of the P1 building block **6** and the P2-P3 dipeptide building block **12**, with the formation of the nitrile warhead at the P1′ position as the final step (Scheme 3). The production of the P1 building block started from the protected amino acid derivative **4**. The amino group of **4** is protected in the form of a carbamate with a *tert*-butoxycarbonyl (Boc) group, which can be cleaved by acidic hydrolysis, and its carboxyl group is protected in the form of a methyl ester, which can be removed under alkaline conditions. In the first step, compound **4** was treated with methanolic ammonia to cleave the methyl ester to give amide **5**, which was Boc-deprotected with hydrochloric acid to form the hydrochloride salt **6**. In parallel, *N*-Boc-*t*-butylalanine (or *N*-Boc-3-methylvaline) (**7**) as the carboxylic acid reactant and compound **8** as the amine reaction partner were coupled to form dipeptide **9**, using diisopropylethylamine (DIEA), as the base and *O*-(7-azabenzotriazol-1-yl)-*N*,*N*,*N’*,*N’*-tetramethyluronium hexafluorophosphate (HATU) as coupling agent; the conditions used are common in peptide chemistry. The methyl ester group of **9** was cleaved with LiOH to give compound **10**, the Boc group was then removed from the *N*-terminus with HCl to give compound **11**. Ethyl trifluoracetate was used to convert the NH_2_ group of **11** to acetamide. The obtained compound **12** containing a free carboxyl group was ready for coupling with compound **6**. Peptide coupling was performed using DIEA as base, 1-[3-(dimethylamino)propyl]-3-ethylcarbodiimide hydrochloride (EDCI) as coupling agent, in the presence of 2-hydroxypyridine 1-oxide (HOPO), which served to suppress the racemization. The inner salt methyl-*N*-(triethylammoniosulfonyl)carbamate is the so-called Burgess reagent, usually used to convert amide groups into nitriles by dehydration. Here, in the last step, the P1′ nitrile group was formed by using Burgess reagent. As a result of the work-up procedure, the product is isolated as a methyl *tert*-butyl ether (MTBE) solvate. Nirmarelvir was obtained with an overall yield of 49% by the synthetic route shown in Scheme 4 [4,23,26].

Of note, compound **8**, a bicyclic proline methyl ester derivative containing three chiral centers, is a key building unit in the synthesis of nirmatrelvir. Since **8** is also a building block of boceprevir, many different synthetic routes have been described for it [27].

Another synthetic method (Scheme 4) was reported by Zhao et al. This is a much shorter and simpler reaction route, consisting of only two amide couplings and a deprotection step starting from **8**, and it also gives free nirmatrelvir instead of MTBE solvate. The overall yield of the presented synthesis steps was 60%, but it should be noted that the synthesis of building blocks **14** and **16** has not been reported [28].

**Scheme 5:**
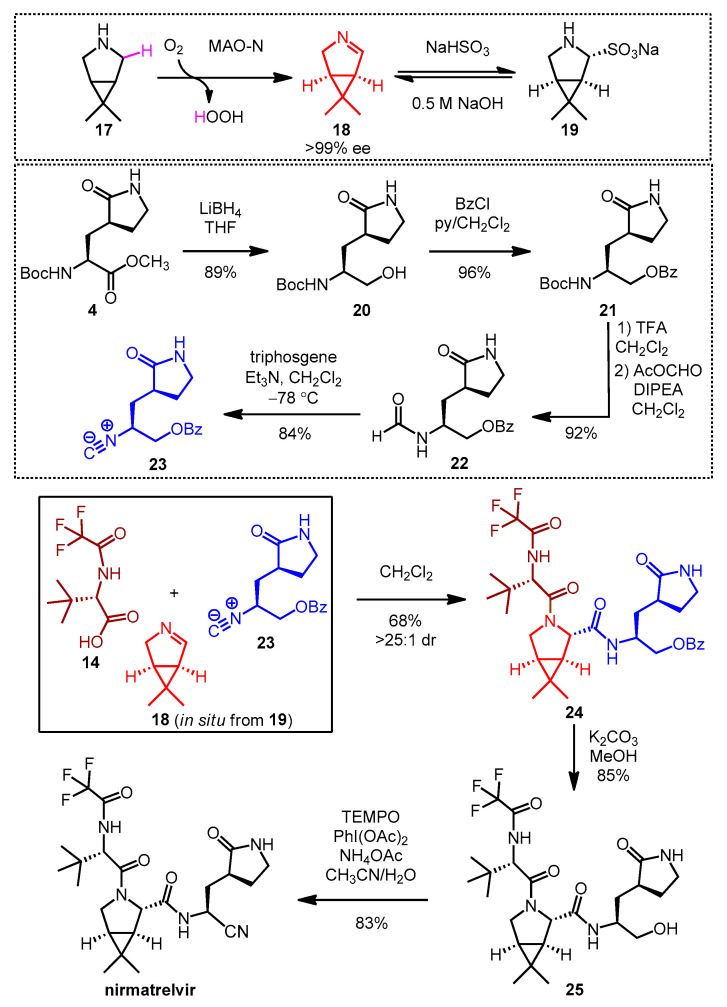
Multicomponent synthesis of nirmatrelvir. (MAO-N: monoamine oxidase N, ee: enantiomeric excess, THF: tetrahydrofuran, BzCl: benzoyl chloride, py: pyridine, TFA: trifluoroacetic acid, DIPEA: disopropylethylamine, Et_3_N: triethylamine, dr: diastereomeric ratio, TEMPO: (2,2,6,6-tetramethylpiperidine-1-yl)oxyl, PhI(OAc)_2_: (diacetoxyiodo)benzene).

In 2023, Ruijter, Turner and co-workers reported a remarkable new synthetic approach to nirmatrelvir based on a highly diastereoselective Ugi-type three-component reaction (Scheme 5) [29]. One of the key building blocks, the chiral bicyclic imine **18**, was prepared by enantioselective oxidative desymmetrization of meso-pyrrolidine **17** with monoamine oxidase N (MAO-N) [30]. Due to the high volatility of **18**, it was isolated in the form of its crystalline bisulfite adduct **19** [31], from which the free imine **18** was generated in situ for the three-component reaction by basic treatment. The isocyanide building block **23** was prepared from the known Boc-protected amino ester **4**. To ensure the appropriate stability and reactivity of isocyanide **23**, the C-terminus of **4** was converted to the protected primary alcohol **21** in two steps including reduction (**20**) and benzoylation. Boc deprotection followed by immediate formylation resulted in formamide **22**, from which cyanide **23** was obtained by dehydration with triphosgene. Ugi-type reaction [32] of the commercially available carboxylic acid **14** with the in situ prepared imine (**18**) and isocyanide (**23**) afforded the nirmatrelvir core **24** in 68% yield and high diastereoselectivity. After debenzoylation, the oxidative conversion of the C-terminal primary alcohol of **25** to a nitrile was performed in a one-pot process, combining PhI(OAc)_2_/TEMPO-mediated oxidation with ammonium acetate as the nitrogen source. This multicomponent synthesis proceeded in six steps yielding nirmatrelvir with an overall yield of 46%.

### 2.4. Synthesis and SAR Study of Nirmatrelvir Analogs

**Figure 4:**
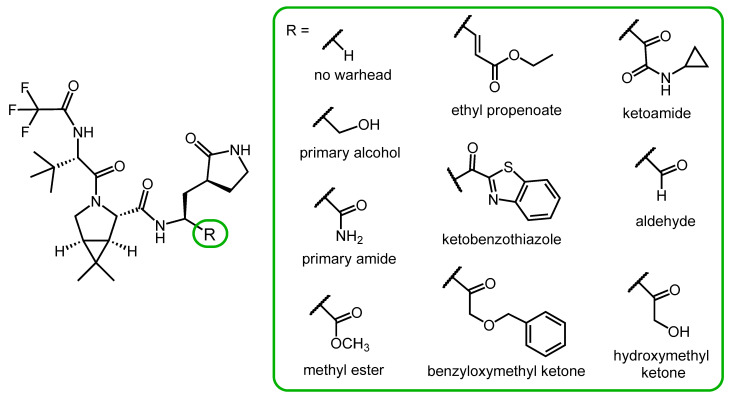
Nirmatrelvir derivatives with alternative warheads.

Chia and co-workers synthesized a small library of nirmatrelvir analogs with different P1′ moieties (Figure 4) to study the role of the warhead in antiviral activity. The compounds were tested for their enzyme inhibitory activity against the 3CLpro (M^pro^) protease of SARS-CoV-2 and hCoV 229E (a human coronavirus that causes the common cold), and their antiviral activity against hCoV 229E. Derivatives without a warhead and with primary alcohol, primary amide, or methyl ester warheads were ineffective.

Derivatives containing ethyl propenoate, ketobenzothiazole, benzyloxymethyl ketone, ketoamide and aldehyde warheads showed similar or better M^pro^ inhibitory effect than nirmatrelvir. However, in a cell-based assay, their antiviral activity against hCoV 229E was inferior to nirmatrelvir, probably due to their lower cell penetration ability.

In this compound library, a derivative with a hydroxymethyl ketone warhead was the most potent one, exerting stronger protease inhibitory activity and similar anti-hCoV activity to nirmatrevil. However, a serious limitation of the study is that the cell-based antiviral tests were only performed with the hCoV 229 coronavirus, not with SARS-CoV-2 [33].

### 2.5. Novel Covalent and Non-Covalent Inhibitors of SARS-CoV-2 M^pro^

**Figure 5:**
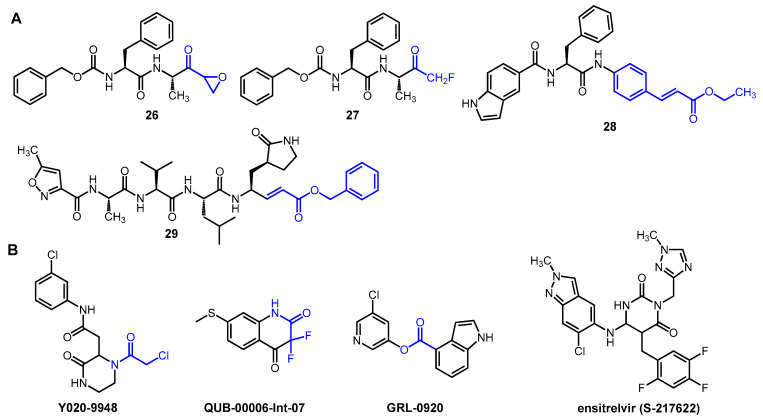
Selected examples of novel peptidic (**A**) and non-peptidic (**B**) inhibitors of M^pro^. Electrophilic warheads in covalent inhibitors are highlighted in blue.

Several other peptidomimetic inhibitors of SARS-CoV-2 M^pro^ (Figure 5A) have been developed with different warheads, including an epoxide ring (**26**), a fluoromethyl group (**27**), a cinnamic ester (**28**) and a vinyl ester (**29**). In the latter two compounds, the α,β-unsaturated ester warhead acts as a Michael acceptor, reacting with the thiolate group of M^Pro^ Cys145, thus forming an irreversible covalent adduct with the enzyme [6,34,35].

Interesting non-peptidic inhibitors were also identified (Figure 5B), such as the commercially available piperazine-2 derivative Y020-9948 with an α-chloroacetamide warhead. Compound QUB-00006-Int-07 was developed based on in silico studies. It contains an α,α-difluoroamide group in a benzene-fused six-membered ring. Esters, such as GRL-0920, also showed significant enzyme inhibitory activity due to the electrophilic nature of the ester group, which makes it a suitable warhead [6].

Non-covalent inhibitors represent an attractive alternative in the development of anti-coronavirus agents. They do not have an electrophilic warhead, so they only form secondary interactions with the active site, such as H-bonds, hydrophobic stacking, and van der Waals forces. For this reason, they generally show lower reactivity but better selectivity than covalent inhibitors [34]. An important representative of non-covalent inhibitors of SARS-CoV-2 M^pro^ is ensitrelvir (S-217622). This compound fits into the S1 and S2 sub-pockets of the active site, where it forms H-bonding and π-π interactions with the enzyme. Ensitrelvir is approved for COVID-19 in Japan and marketed under the brand name Xocova. Despite its proven efficacy, it is insufficient for hard endpoints such as mortality or hospitalization, which, along with other problems (e.g., the occurrence of resistance), may limit its future use [36].

## 3. Ritonavir as a Pharmacokinetic Enhancer

### 3.1. Structure, Enzyme Inhibitory Activity and Drug–Drug Interactions of Ritonavir

**Figure 6:**
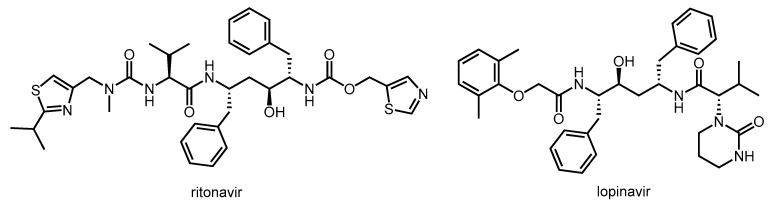
Structure of ritonavir and lopinavir—a combination tested and failed against SARS-CoV-2 in drug-repurposing.

Ritonavir (Figure 6) was originally developed as an HIV protease inhibitor, but unfortunately, it is very poorly tolerated at an effective antiviral dose (400–500 mg). On the other hand, it turned out that ritonavir at a low dose (~100 mg) can outstandingly inhibit the CYP3A4 enzyme [34,37,38]. CYP3A4 is a member of the cytochrome P450 enzyme superfamily. It is mainly produced in the liver and is responsible for the metabolism of many endogenous and exogenous molecules, including many drugs [39]. Taking advantage of its CYP3A4 inhibitory effect, ritonavir has been used in combination with other HIV PIs (e.g., saquinavir, indinavir, or lopinavir) to prevent the degradation of these pharmacons. In these combinations, ritonavir is used at a low dose (usually 100 mg), in which it has no significant antiviral effect but is relatively well tolerated, while the other PI is used at a higher concentration as an antiviral agent. The advantage of co-administering an antiviral PI with ritonavir is that inhibition of CYP3A4 increases the half-life of the antiviral component, allowing lower doses or less frequent dosing. Both reduce therapy costs and increase patient adherence [37,38]. In this context, it is worth mentioning that in the early stages of the pandemic, the lopinavir/ritonavir combination originally developed against HIV was tested for the treatment of COVID-19, but was found to be ineffective [40].

The role of ritonavir in Paxlovid^TM^ is to enhance the effects of nirmatrelvir by inhibiting its metabolism by CYP3A4. The disadvantage of using ritonavir is the increased risk of drug–drug interactions (DDI). The most important type of potential DDI is when the patient is taking other drugs metabolized by CYP3A4 (e.g., simvastatin or midazolam). In this case, ritonavir may even increase the concentration of the other drug to a toxic level. This can be a serious problem if the other drug used has a narrow therapeutic index (the concentration range in which the drug is effective but not toxic), such as tacrolimus, an immunosuppressive agent. Due to the effect of ritonavir on metabolism, it is worth considering, if possible, temporarily suspending (or replacing with other drugs) the use of drugs that are mainly metabolized by CYP3A4, during treatment with Paxlovid and for 3 days afterwards. However, stopping the regimen does not help with drugs with a long half-life, such as, e.g., the antiarrhythmic drug amiodarone, because its plasma concentration may remain high for a long time after the drug is discontinued [12].

Another type of drug–drug interaction is the use of enzyme-inducing drugs (e.g., phenobarbital or carbamazepine), which can counteract the effects of ritonavir by increasing CYP3A4 activity. The inducer effect may persist even after the administration of the inducer molecule is stopped, therefore, pausing the application does not eliminate this interaction [12,41]. Ritonavir also affects other enzymes and transport proteins, but these are of minor importance during Paxlovid treatment.

**Scheme 6:**
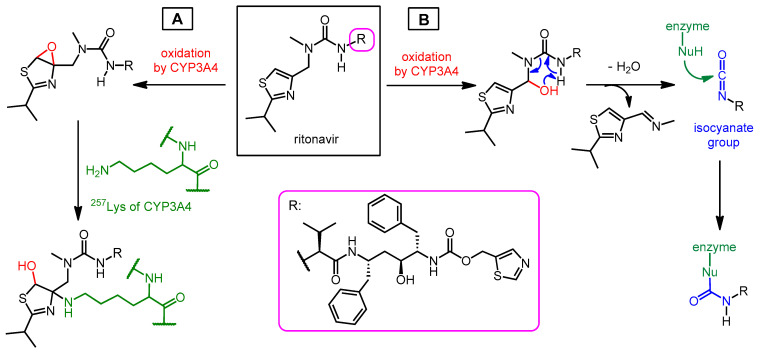
Possible mechanism of the irreversible inactivation of CYP3A4 by ritonavir. Nucleophilic inactivation occurs via either an epoxide (pathway A) or an isocyanate intermediate (pathway B).

Although it is well known that ritonavir is a strong, irreversible inhibitor of the CYP3A4 enzyme, the precise mechanism of inhibition is currently not fully understood. Various mechanisms have been proposed in the literature for the inactivation of CYP3A4, such as (i) coordination of an uncharacterized metabolic intermediate of ritonavir to the heme unit of the enzyme, (ii) ligation of ritonavir to the hem iron of the enzyme, and (iii) covalent binding of a reactive ritonavir intermediate to CYP3A4 apoprotein [42]. There are two proposed mechanisms for the latter covalent attachment (Scheme 6). One possible mechanism is that after oxidation by CYP3A4, an elimination reaction takes place, resulting in an isocyanate derivative (route B) [20]. Isocyanates are carbamoylating agents that can react with nucleophilic groups of proteins (e.g., amino groups). Therefore, ritonavir covalently binds to CYP3A4, rendering it inactive. The restoration of enzyme activity requires the synthesis of new CYP3A4 molecules, which takes time. This is why the inhibitory effect slowly wears off after stopping ritonavir. According to the other proposed mechanism, the double bond of the thiazole ring of ritonavir is oxidized to an epoxide, then the amino group 254Lys of the enzyme opens the epoxide ring, so that ritonavir is covalently bound to the apoprotein (route A) [42,43].

### 3.2. Synthesis of Ritonavir

**Scheme 7:**
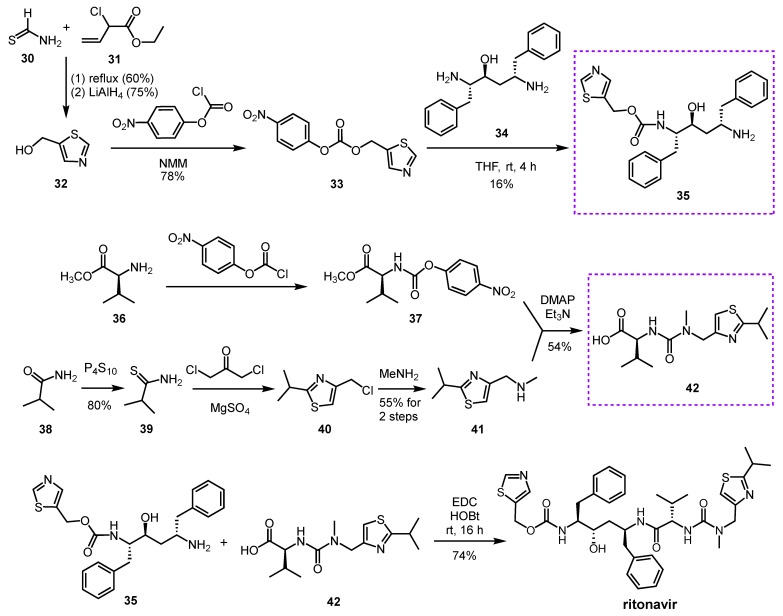
Synthesis of ritonavir. (NMM: N-methylmorpholine, DMAP: 4-dimethylaminopyridine, EDC: 1-ethyl-3-(3-dimethylaminopropyl)carbodiimide, HOBt: 1-hydroxybenzotriazole).

Ritonavir was developed as an HIV protease inhibitor at Abbott Laboratories [44]. The synthesis strategy of ritonavir involved the preparation and coupling of the chiral building blocks of amine **35** and carboxylic acid **42** (Scheme 7) [45,46]. First, cyclocondensation reaction of thioformamide **30** and ethyl 2-chloro-2-chloroacetate **31** followed by reduction with lithium aluminum hydride resulted in 5-hydroxymethylthiazole **32**. By reacting compound **32** with 4-nitrophenylchloroformic acid, carbonate derivative **33** is obtained, which is then combined with chiral diamino alcohol **34** to obtain compound **35**, which contains one of the carbamate groups of ritonavir. There are several methods for the synthesis of the (*S,S,S*)-diamino alcohol (**34**) used, which are not discussed here [46,47]. In a parallel route, valine methyl ester **36** was activated as carbamate (**37**) with 4-nitrophenylchloroformic acid. *i*-Butyramide (**38**) was converted to 2-methylpropanethioamide **39** by oxygen–sulfur exchange with phosphorus pentasulfide. Then, by cyclocondensation of compound **39** with 1,3-dichloroacetone, thiazole derivative **40** is obtained, which is converted into the N-methyl derivative **41** in the reaction with methylamine. Compounds **37** and **41** are coupled in the presence of triethylamine and 4-dimethylaminopyridine bases; the methyl ester is cleaved parallel to the formation of the amide bond, thus obtaining compound **42** with a free carboxyl group. Compounds **35** containing an amino group and **42** containing a carboxyl group were coupled by classical peptide synthesis, using EDC as a coupling agent and HOBt to prevent racemization, to give ritonavir.

## 4. Paxlovid—Application and Activity against Mutant Variants

Paxlovid^TM^ is a product that contains co-packed nirmatrelvir (150 mg/tablet) and ritonavir (100 mg/tablet). The normal dose is 300 mg nirmatrelvir (two tablets) and 100 mg ritonavir (one tablet) two times per day. Nirmatrelvir dosage should be reduced in patients with moderate kidney dysfunction, and its use is not recommended in patients with severe kidney dysfunction. The treatment lasts 5 days [48].

In a phase II/III clinical trial (EPIC-HR = Evaluation of Protease Inhibition for COVID-19 in High-Risk Patients), Paxlovid reduced the combined risk of death and hospitalization related to COVID-19 by 89% compared to the placebo group [49]. A 2022 meta-analysis found that nirmatrelvir/ritonavir was successful in reducing hospitalizations and mortality in patients with COVID-19, but there was no difference between emergency department visits and intensive care unit admissions based on an analysis of 314,353 patient trials. [9] Unfortunately, the EPIC-SR trial, which was designed to test Paxlovid in a standard-risk population, was terminated due to low hospitalization/death rates in the standard-risk population.

Paxlovid was approved for emergency use authorization by the USA in 2021, for the treatment of patients with mild/moderate COVID-19 with high risk of progression to severe disease, with no requirement of oxygen supply. It gained conditional authorization in 2021 in the UK and in 2022 in the EU [48].

Nirmatrelvir has proven to be highly active against the Omicron variant of SARS-CoV-2 and its sub-variants. However, there are already circulating strains with mutations that may give the virus some level of resistance to nirmatrelvir [50,51]. Fortunately, such known mutations are not very common. In a study based on the GISAID database, among more than 13 million sequences, the occurrence of resistance-causing mutations was 0.5%, and no increasing trend was observed; however, there are strains in which certain mutations are dominant [52]. Most of the omicron subvariants are still sensitive to nirmatrelvir, but it is important to monitor the emergence of new potentially resistant strains [53].

## 5. Conclusions

Although there are several effective and safe vaccines against COVID-19, this virus is unlikely to disappear anytime soon, so there is a need for effective therapeutic agents, especially oral medicines that people can take at home. There are open questions about Paxlovid, e.g., the therapeutic advantage for vaccinated and standard-risk patients, or the phenomenon of rebound, which means that symptoms reappear in some patients after the end of therapy [54,55,56]. Nevertheless, Paxlovid is a valuable antiviral agent against SARS-CoV-2 with high efficacy and safety. At the same time, despite the success of the nirmatrelvir–ritonavir combination, research into alternative M^Pro^ inhibitors should not be stopped; fortunately, research efforts aimed at developing novel M^Pro^ inhibitors are ongoing [34], as was briefly presented in Section 2.5.

The development of pan-coronavirus antivirals, based on the screening of natural compounds or the design of new molecules, is emerging as a new strategy to combat potential future pandemics. Fusion inhibitors are typically considered pan-coronavirus agents that target the heptad repeat 1 (HR-1) domain of the spike protein S2 subunit. Fusion inhibitors can effectively inhibit the infection of SARS-CoV-2 variants and other human coronaviruses, their broad-spectrum effect is based on the fact that the spike protein (S protein) responsible for viral entry plays a key role in viral infections, and the HR-1 domain is highly conserved region between coronaviruses [57,58,59].

## Data Availability

Data are contained within the article.

## References

[1] Sharma A., Gupta S.P., Gupta S.P. (2017). Fundamentals of Viruses and Their Proteases. Viral Proteases and Their Inhibitors.

[2] Testa B., Mayer J.M. (2003). Hydrolysis in Drug and Prodrug Metabolism Chemistry Biochemistry and Enzymology.

[3] Majerová T., Konvalinka J. (2022). Viral proteases as therapeutic targets. Mol. Asp. Med..

[4] Owen D.R., Allerton C.M.N., Anderson A.S., Aschenbrenner L., Avery M., Berritt S., Boras B., Cardin R.D., Carlo A., Coffmann K.J. (2021). An oral SARS-CoV-2 Mpro inhibitor clinical candidate for the treatment of COVID-19. Science.

[5] Mengist H.M., Dilnessa T., Jin T. (2021). Structural Basis of Potential Inhibitors Targeting SARS-CoV-2 Main Protease. Front. Chem..

[6] Citarella A., Dimasi A., Moi D., Passarella D., Scala A., Piperno A., Micale N. (2023). Recent Advances in SARS-CoV-2 Main Protease Inhibitors: From Nirmatrelvir to Future Perspectives. Biomolecules.

[7] Wen W., Chen C., Tang J., Wang C., Zhou M., Cheng Y., Zhou X., Wu Q., Zhang X., Feng Z. (2022). Efficacy and safety of three new oral antiviral treatment (molnupiravir, fluvoxamine and Paxlovid) for COVID-19: A meta-analysis. Ann. Med..

[8] Pesko B., Deng A., Chan J.D., Neme S., Dhanireddy S., Jain R. (2022). Safety, and tolerability of paxlovid (nirmatrelvir/ritonavir) in high risk patients. Clin. Infect. Dis..

[9] Amani B., Amani B. (2023). Efficacy and safety of nirmatrelvir/ritonavir (Paxlovid) for COVID-19: A rapid review and meta-analysis. J. Med. Virol..

[10] Paltra S., Conrad T. (2023). Effectiveness of Paxlovid—A review. medRxiv.

[11] Zhu C.-T., Yin J.-Y., Chen X.-H., Liu M., Yang S.-G. (2023). Appraisal of evidence reliability and applicability of Paxlovid as treatment for SARS-COV-2 infection: A systematic review. Rev. Med. Virol..

[12] Gerhart J., Cox D.S., Singh R.S.P., Chan P.L.S., Rao R., Allen R., Shi H., Masters J.C., Damle B. (2024). A Comprehensive Review of the Clinical Pharmacokinetics, Pharmacodynamics, and Drug Interactions of Nirmatrelvir/Ritonavir. Clin. Pharmacokinet..

[13] de Leuw P., Stephan C. (2018). Protease inhibitor therapy for hepatitis C virus-infection. Expert Opin. Pharmacother..

[14] Halfon P., Locarnini S. (2011). Hepatitis C virus resistance to protease inhibitors. J. Hepatol..

[15] Schechter I., Berger A. (1967). On the size of the active site in proteases. I. Papain. Biochem. Biophys. Res. Commun..

[16] Lv Z., Chu Y., Wang Y. (2015). HIV protease inhibitors: A review of molecular selectivity and toxicity. HIV/AIDS (Auckl.).

[17] Ghosh A.K., Osswald H.L., Prato G. (2016). Recent Progress in the Development of HIV-1 Protease Inhibitors for the Treatment of HIV/AIDS. J. Med. Chem..

[18] Hoffman R.L., Kania R.S., Brothers M.A., Davies J.F., Ferre R.A., Gajiwala K.S., He M., Hogan R.J., Kozminski K., Li L.Y. (2020). Discovery of ketone-based covalent inhibitors of coronavirus 3CL proteases for the potential therapeutic treatment of COVID-19. J. Med. Chem..

[19] Lee N., Hui D., Wu A., Chan P., Cameron P., Joynt G.M., Ahuja A., Yung M.Y., Leung C.B., To K.F. (2003). A major outbreak of severe acute respiratory syndrome in Hong Kong. N. Engl. J. Med..

[20] Menéndez J.C. (2022). Approaches to the Potential Therapy of COVID-19: A General Overview from the Medicinal Chemistry Perspective. Molecules.

[21] Veber D.F., Johnson S.R., Cheng H., Smith B.R., Ward K.W., Kopple K.D. (2002). Molecular Properties That Influence the Oral Bioavailability of Drug Candidates. J. Med. Chem..

[22] Chia C.S.B. (2022). Novel Nitrile Peptidomimetics for Treating COVID-19. Med. Chem. Lett..

[23] Joyce R.P., Hu V.W., Wang J. (2022). The history, mechanism, and perspectives of nirmatrelvir (PF-07321332): An orally bioavailable main protease inhibitor used in combination with ritonavir to reduce COVID-19-related hospitalizations. Med. Chem. Res..

[24] Li J., Lin C., Zhou X., Zhong F., Zheng P., Yang Y., Zhang Y., Yu B., Fan X., McCormick P.J. (2022). Structural Basis of the Main Proteases of Coronavirus Bound to Drug Candidate PF-07321332. Virol. J..

[25] Ngo S.T., Nguyen T.H., Tung N.T., Mai B.K. (2022). Insights into the binding and covalent inhibition mechanism of PF-07321332 to SARS-CoV-2 Mpro. RSC Adv..

[26] Marzi M., Vakil M.K., Bahmanyar M., Zarenezhad E. (2022). Paxlovid: Mechanism of Action, Synthesis, and In Silico Study. Biomed. Res. Int..

[27] Kallam S.R., Eda V.R., Sen S., Datrika R., Rapolu R.K., Khobare S., Gajare V., Banda M., Khan R.A.R., Singh M. (2017). A diastereoselective synthesis of boceprevir’s gem-dimethyl bicyclic [3.1.0] proline intermediate from an insecticide ingredient cis-cypermethric acid. Tetrahedron.

[28] Zhao Y., Fang C., Zhang Q., Zhang R., Zhao X., Duan Y., Wang H., Zhu Y., Feng L., Zhao J. (2022). Crystal structure of SARS-CoV-2 main protease in complex with protease inhibitor PF-07321332. Protein Cell.

[29] Preschel H.D., Otte R.T., Zhuo Y., Ruscoe R.E., Burke A.J., Kellerhals R., Horst B., Hennig S., Janssen E., Green A.P. (2023). Multicomponent Synthesis of the SARS-CoV-2 Main Protease Inhibitor Nirmatrelvir. J. Org. Chem..

[30] Köhler V., Bailey K.R., Znabet A., Raftery J., Helliwell M., Turner N.J. (2010). Enantioselective Biocatalytic Oxidative Desymmetrization of Substituted Pyrrolidines. Angew. Chem. Int. Ed..

[31] Flores-Reyes J.C., Islas-Jácome A., González-Zamora E. (2021). The Ugi three-component reaction and its variants. Org. Chem. Front..

[32] Li T., Liang J., Ambrogelly A., Brennan T., Gloor G., Huisman G., Lalonde J., Lekhal A., Mijts B., Muley S. (2012). Efficient, Chemo-enzymatic Process for Manufacture of the Boceprevir Bicyclic [3.1.0] Proline Intermediate Based on Amine Oxidase-Catalyzed Desymmetrization. J. Am. Chem. Soc..

[33] Vankadara S., Dawson M.D., Fong J.Y., Oh Q.Y., Ang Q.A., Liu B., Chang H.Y., Koh J., Koh X., Tan Q.W. (2022). A Warhead Substitution Study on the Coronavirus Main Protease Inhibitor Nirmatrelvir. Med. Chem. Lett..

[34] Citarella A., Moi D., Pedrini M., Pérez-Peña H., Pieraccini S., Dimasi A., Stagno C., Micale N., Schirmeister T., Sibille G. (2023). Synthesis of SARS-CoV-2 Mpro inhibitors bearing a cinnamic ester warhead with in vitro activity against human coronaviruses. Org. Biomol. Chem..

[35] Ashraf-Uz-Zaman M., Chua T.K., Li X., Yao Y., Moku B.K., Mishra C.B., Avadhanula V., Piedra P.A., Song Y. (2024). Design, Synthesis, X-ray Crystallography, and Biological Activities of Covalent, Non-Peptidic Inhibitors of SARS-CoV-2 Main Protease. ACS Infect. Dis..

[36] Ferraro S., Convertino I., Cappello E., Valdiserra G., Bonasoa M., Tuccori M. (2024). Lessons learnt from the preclinical discovery and development of ensitrelvir as a COVID-19 therapeutic option. Expert Opin. Drug Discov..

[37] Cooper C.L., van Heeswijk R.P.G., Gallicano K., Cameron D.W. (2003). A Review of Low-Dose Ritonavir in Protease Inhibitor Combination Therapy. Clin. Infect. Dis..

[38] Cvetkovic R.S., Goa K.L. (2003). Lopinavir/Ritonavir A Review of its Use in the Management of HIV Infection. Drugs.

[39] Klein K., Zanger U.M. (2013). Pharmacogenomics of cytochrome P450 3A4: Recent progress toward the “missing heritability” problem. Front. Genet..

[40] Patel T.K., Patel P.B., Barvaliya M., Saurabh M.K., Bhalla H.L., Khosla P.P. (2021). Efficacy and safety of lopinavir-ritonavir in COVID-19: A systematic review of randomized controlled trials. J. Infect. Public Health.

[41] Marzolini C., Kuritzkes D.R., Marra F., Boyle A., Gibbons S., Flexner C., Pozniak A., Boffito M., Waters L., Burger D. (2022). Recommendations for the Management of Drug-Drug Interactions Between the COVID-19 Antiviral Nirmatrelvir/Ritonavir (Paxlovid) and Comedications. Clin. Pharmacol. Ther..

[42] Loos N.H.C., Beijnen J.H., Schinkel A.H. (2022). The Mechanism-Based Inactivation of CYP3A4 by Ritonavir: What Mechanism?. Int. J. Mol. Sci..

[43] Rock B.M., Hengel S.M., Rock D.A., Wienkers L.C., Kunze K.L. (2014). Characterization of ritonavir-mediated inactivation of cytochrome P450 3A4. Mol. Pharmacol..

[44] Kempf D.J., Sham H.L., Marsh K.C., Flentge C.A., Betebenner D., Green B.E., McDonald E., Vasavanonda S., Saldivar A., Wideburg N.E. (1998). Discovery of ritonavir, a potent inhibitor of HIV protease with high oral bioavailability and clinical efficacy. J. Med. Chem..

[45] Ghosh A.K., Bilcer G., Schiltz G. (2001). Syntheses of FDA Approved HIV Protease Inhibitors. Synthesis.

[46] Kempf D.J., Marsh K.C., Fino L.C., Bryant P., Craig-Kennard A., Sham H.L., Zhao C., Vasavanonda S., Kohlbrenner W.E., Wideburg N.E. (1994). Design of Orally Bioavailable, Symmetry-Based Inhibitors of HIV Protease. Bioorg. Med. Chem..

[47] Ghosh A.K., McKee S.P., Thompson W.J., Darke P.L., Zugay J.C. (1993). Potent HIV-1 Protease Inhibitors: Stereoselective Synthesis of a Dipeptide Mimic. J. Org. Chem..

[48] Lamb Y.N. (2022). Nirmatrelvir Plus Ritonavir: First Approval. Drugs.

[49] Hammond J., Leister-Tebbe H., Gardner A., Abreu P., Bao W., Wisemandle W., Baniecki M.L., Hendrick V.M., Damle B., Simón-Campos A. (2022). Oral Nirmatrelvir for High-Risk, Nonhospitalized Adults with Covid-19. N. Eng. J. Med..

[50] Hu Y., Lewandowski E.M., Tan H., Zhang X., Morgan R.T., Zhang X., Jacobs L.M.C., Butler S.G., Gongora M.V., Choy J. (2023). Naturally occurring mutations of SARS-CoV-2 main protease confer drug resistance to nirmatrelvir. ACS Cent. Sci..

[51] Mótyán J.A., Mohamed Mahdi M., Hoffka G.Y., Tőzsér J. (2022). Potential Resistance of SARS-CoV-2 Main Protease (Mpro) against Protease Inhibitors: Lessons Learned from HIV-1 Protease. Int. J. Mol. Sci..

[52] Ip J.D., Chu A.W., Chan W., Leung R.C., Abdullah S.M.U., Sun Y., To K.K. (2023). Global prevalence of SARS-CoV-2 3CL protease mutations associated with nirmatrelvir or ensitrelvir resistance. eBioMedicine.

[53] Chatterjee S., Bhattacharya M., Dhama K., Lee S., Chakraborty C. (2023). Resistance to nirmatrelvir due to mutations in the Mpro in the subvariants of SARS-CoV-2 Omicron: Another concern?. Mol. Ther. Nucleic Acids.

[54] Epling B.P., Rocco J.M., Boswell K.L., Laidlaw E., Galindo F., Kellogg A., Das S., Roder A., Ghedin E., Kreitman A. (2022). COVID-19 redux: Clinical, virologic, and immunologic evaluation of clinical rebound after nirmatrelvir/ritonavir. medRxiv.

[55] Antonelli G., Focosi D., Turriziani O., Tuccori M., Brandi R., Fillo S., Ajassa C., Lista F., Mastroianni C.M. (2022). Virological and clinical rebounds of COVID-19 soon after nirmatrelvir/ritonavir discontinuation. Clin. Microbiol. Infect..

[56] Focosi D., McConnell S., Shoham S., Casadevall A., Maggi F., Antonelli G. (2023). Nirmatrelvir and COVID-19: Development, pharmacokinetics, clinical efficacy, resistance, relapse, and pharmacoeconomics. Int. J. Antimicrob. Agents.

[57] Mori M., Quaglio D., Calcaterra A., Ghirga F., Sorrentino L., Cammarone S., Fracella M., D’Auria A., Frasca F., Criscuolo E. (2023). Natural Flavonoid Derivatives Have Pan-Coronavirus Antiviral Activity. Microorganisms.

[58] Wu L., Zheng A., Tang Y., Chai Y., Chen J., Cheng L., Hu Y., Qu J., Lei W., Liu W.J. (2023). A pan-coronavirus peptide inhibitor prevents SARS-CoV-2 infection in mice by intranasal delivery. Sci. China Life Sci..

[59] Lan Q., Wang L., Jiao F., Lu L., Xia S., Jiang S. (2023). Pan-coronavirus fusion inhibitors to combat COVID-19 and other emerging coronavirus infectious diseases. J. Med. Virol..

